# A Novel Role of Connexin 40-Formed Channels in the Enhanced Efficacy of Photodynamic Therapy

**DOI:** 10.3389/fonc.2019.00595

**Published:** 2019-07-09

**Authors:** Deng-Pan Wu, Li-Ru Bai, Yan-Fang Lv, Yan Zhou, Chun-Hui Ding, Si-Man Yang, Fan Zhang, Yuan-Yuan Wang, Jin-Lan Huang, Xiao-Xing Yin

**Affiliations:** ^1^Jiangsu Key Laboratory of New Drug Research and Clinical Pharmacy, Pharmacy School of Xuzhou Medical University, Xuzhou, China; ^2^Department of Pharmacology, Pharmacy School of Xuzhou Medical University, Xuzhou, China; ^3^Department of Pharmacy, Wuxi Ninth Affiliated Hospital of Suzhou University, Wuxi, China; ^4^Scientific Research Center of Traditional Chinese Medicine, Guangxi University of Chinese Medicine, Nanning, China

**Keywords:** Connexin 40, channel, photodynamic therapy, reactive oxygen species, calcium

## Abstract

Despite responses to initial treatment of photodynamic therapy (PDT) being promising, a recurrence rate exists. Thus, finding novel therapeutic targets to enhance PDT efficacy is an urgent need. Reports indicate that connexin (Cx) 40 plays an important role in tumor angiogenesis and growth. However, it is unknown whether Cx40-composed channels have effects on PDT efficacy. The study uniquely demonstrated that Cx40-formed channels could enhance the phototoxicity of PDT to malignant cells *in vitro* and *in vivo*. Specifically, Cx40-formed channels at high cell density could increase PDT photocytotoxicity. This action was substantially restricted when Cx40 expression was not induced or Cx40 channels were restrained. Additionally, the presence of Cx40-composed channels enhanced the phototoxicity of PDT in the tumor xenografts. The above results indicate that enhancing the function of Cx40-formed channels increases PDT efficacy. The enhancement of PDT efficacy mediated by Cx40 channels was related with intracellular pathways mediated by ROS and calcium pathways, but not the lipid peroxide-mediated pathway. This work demonstrates the capacity of Cx40-mediated channels to increase PDT efficacy and suggests that therapeutic strategies designed to maintain or enhance Cx40 expression and/or channels composed by Cx40 may increase the therapeutic efficacy of PDT.

## Introduction

Photodynamic therapy (PDT) is a novel cancer treatment and acts primarily via the generation of reactive oxygen species (ROS) through the irradiation of photosensitized cells, resulting in the destruction of subcellular sites in which the photosensitizer has localized (1). PDT has been reported to treat malignancies of skin, brain, esophagus, lung and prostate (2). The photosensitizer Photofrin was firstly approved by the Food and Drug Administration in 1995, and Photofrin-mediated PDT has been utilized to treat patients with completely or partially obstructing esophageal cancer (3). Although the responses of the initial treatment of PDT are promising, there is a recurrence rate (4). Therefore, it would be therapeutically beneficial to explore novel targets for the enhancement of PDT efficacy.

Connexin (Cx) channels, which are composed of connexins (Cxs), mediate direct intercellular transfer of molecules and/or electrical charge (5). Six Cxs compose a hemichannel that docks to a counterpart in a neighboring cell to form a GJ channel (6). These Cx channels allow intercellular transfer of signaling molecules cGMP, cAMP and phosphoinositides, thereby playing vital roles in a variety of processes including cellular differentiation, proliferation and tumor suppression (7). Recently, the role of Cx channels in therapeutic usage has generated considerable interest. For example, Cx channels have the ability to improve the cytotoxicity of chemotherapeutics and ionizing radiation (8, 9). Such an action has also been found when prostate cancer cells were treated with tumor necrosis factor-α (TNF-α), in which Cxs-based channels enhance the TNF-α-induced apoptosis (10).

Based on the above findings, it can be inferred that during the apoptotic or necrotic processes, molecules called “death signal” existing in one cell can penetrate via Cx channels to neighboring cells. This is referred to as a “bystander effect.” It should be noted that intracellular ROS and Ca^2+^, which are responsible for the photodamage of malignant cells, can spread via Cx channels to untreated neighboring cells (11, 12). Studies have shown that lipid peroxides including 4-hydroxynonenal (4-HNE) and ceramide contribute to the damage to the cells after PDT (13, 14). 4-HNE and ceramide have possibilities to diffuse through Cx channels since their molecular weights are less than the upper limit of molecules penetrating via Cx channels. In consideration of the above findings, the diffusion of these “death signals” via Cx channels might impact PDT phototoxicity.

Cx40 is one of the Cx family members forming Cx channels. It has been established that Cx40 plays an important role in tumor angiogenesis, growth and migration (15, 16). However, the role of Cx40-composed channels in cancer treatment including PDT has been less reported. Thus, the present study was designed to explore the role of Cx40 channels in the efficacy of Photofrin-mediated PDT and its potential mechanisms. The results showed that Cx40 channels could increase the phototoxicity of transfected HeLa cells induced by PDT. This phototoxic action was associated with the increased intracellular ROS production and enhanced amounts of intracellular Ca^2+^ by Cx40 channels. This study indicates that maintenance or even transitory enhancement of Cx40 expression and Cx40-formed channels is a beneficial strategy for increasing the therapeutic effect of PDT. On the contrary, factors inhibiting the function of Cx40-composed channels may cause the insensitivity of malignancies to PDT, resulting in a significant decline in therapeutic effect of PDT.

## Materials and Methods

### Materials

Photofrin® (75 mg/vial) was obtained from Union Med. Group Limited Company, Hong Kong, China. Doxycycline (Dox) was supplied by Abcam. Puromycin was provided by Abcam. Cell culture reagents were obtained from Life Technology. Other reagents were all supplied by Sigma-Aldrich unless otherwise stated.

### Cell Culture and Photofrin Treatment

The HeLa cell line used in the study was transfected with tetracycline operon (Tet-on) inducible gene expression system as described in our previous study (11). The expression of Cx40 can be induced with 1 μg/mL Dox for 48 h. Cells were cultured in DMEM supplemented with 10% fetal bovine serum (FBS) and 2 μg/mL puromycin. Stock Photofrin solution (10 mg/mL) was prepared with 5% dextrose solution in the dark and was diluted to variable concentrations by DMEM when used. Photosensitizer treatment was performed as previously described (11). Briefly, after 4 h incubation with Photofrin, cells were irradiated at 630 nm (20 mW/cm^2^ for 2 J/cm^2^) and then cultured in complete DMEM medium without photosensitizer for 24 h in the dark until Photofrin phototoxicity was measured using CCK-8 assay. 18α-GA (final concentration: 10 μM) was exposed to the cells 2 h before Photofrin treatment.

### Photosensitivity Assessment

For investigating the role of Cx40-formed channels in PDT photosensitivity, after cells were treated with Dox in one culture flask to induce Cx40 expression and channel formation, they were seeded in 96-well plates under low and high cell density conditions. At high cell density, 3 × 10^4^ cells/cm^2^ cells were seeded with 70%-100% confluence. Under such circumstances, a single cell was allowed to contact 3–5 others and provided substantial opportunity for channel formation when treated with Photofrin. For the low-cell density, 3 × 10^3^ cells/cm^2^ cells were seeded into 96-well plates. Under such conditions, cells did not have the opportunity to contact each other and channel formation was not allowed. After PDT, CCK-8 solution (Dojindo Molecular Technologies, Japan) was added and incubated with cells for 2 h. The OD values were measured to determine cell survival using an Enzyme-labeling instrument.

### *In vivo* Photosensitivity

For determining the role of Cx40 channels in photosensitivity *in vivo*, BALB/c-nude mice were transplanted with HeLa cells expressing Cx40 according to our previous study (12). Briefly, the mice (16–22 g) bearing xenografts were treated with sucrose water containing Dox (0.2 mg/mL Dox, 5% sucrose) during the experiment. When the tumor xenografts were grown to 100–300 mm^3^, 2.5 mg/kg Photofrin or 0.5% sterile dextrose (control) was intravenously administrated; the mice were kept in darkness for 24 h and then the tumors were irradiated (630 nm, 75 mW/cm^2^, 135 J/cm^2^). After PDT, the xenograft volume (V) was determined based on the equation V = (L × W^2^) ×0.5, where L is the longest axis of xenograft and W is the axis perpendicular to L. The relative tumor volume (RTV) of each xenograft was calculated according to the equation RTV = volume of each time point/volume prior to treatment. After euthanasia, the xenografts were excised and weighted. The weight inhibition rate was defined by the following equation: Tumor weight inhibition rate = (1-mean tumor weight of treated groups/mean tumor weight of control group) ×100%.

### Western Blot Assay

The western blot assay was utilized as described in previous studies (17, 18). Briefly, after the separation of protein samples, samples were transferred to nitrocellulose transfer membrane (Excell Bio, China). The dilution of Cx40 antibody and immunoglobulin-conjugated anti-rabbit was 1:1000 and 1:10000 respectively.

### “Parachute” Dye-Coupling Assay

The assay was utilized for measuring the function of Cx40 channels as previously described (11, 12). In brief, after donor and receiver cells were at confluence, calcein-acetoxymethyl ester (5 μM) was added and incubated with donor cells for 30 min, and then donor cells were trypsinized and seeded to the receiver cells at a 1:150 donor/receiver ratio for 4 h at 37°C, allowing donor cells to attach receiver cells to form channels. The number of receiver cells containing dye per donor cell was counted and normalized to the control group.

### ROS and Ca^2+^ Detection Using Flow Cytometer

The determination of intracellular ROS and Ca^2+^ concentrations was performed according to our previous study (11). In brief, after incubation with or without photosensitizer for 4 h, cells at confluence were incubated with 20 mM dichlorodihydrofluorescein diacetate (DCFH-DA, Beyotime, China) and 5 μM Fluo-3-Am (Beyotime, China) for 1 h for detection of ROS and Ca^2+^, respectively. For detection of intracellular ROS, 30 min after irradiation, cells were collected after trypsinization and resuspended in PBS. With regard to intracellular Ca^2+^ detection, freshly prepared Ca^2+^ balanced salt solution (2 mMMgCl_2_, 10 mM HEPES, 140 mM NaCl, 2 mMCaCl_2_ and 2.8 mM KCl, pH 7.2) or Ca^2+^ free BBS (2.8 mM KCl, 140 mM NaCl and 10 mM HEPES, pH 7.2) was added and illumined. After PDT, cell suspension was prepared after cells were trypsinized and collected. The intracellular concentrations of Ca^2+^ and ROS of Dox-treated and -untreated cells were determined by flow cytometer and normalized to Dox-treated and -untreated cells in control group, respectively.

### 4-HNE and Ceramide Detection

4-HNE and ceramide were measured using ELISA according to our previous study (11). Briefly, cells at 70–100% confluence were incubated with or without photosensitizer for 4 h. One hour after PDT, cells were collected and intracellular levels of 4-HNE and ceramide were determined by ELISA kit (Shanghai Enzyme-linked Biotechnology Co., Ltd, China). The levels of 4-HNE and ceramide of Dox-treated and -untreated cells in the Photofrin group were normalized to Dox-treated and -untreated cells in the control group, respectively.

## Results

### PDT Phototoxicity Depends on the Cell Density

**Figure 1:**
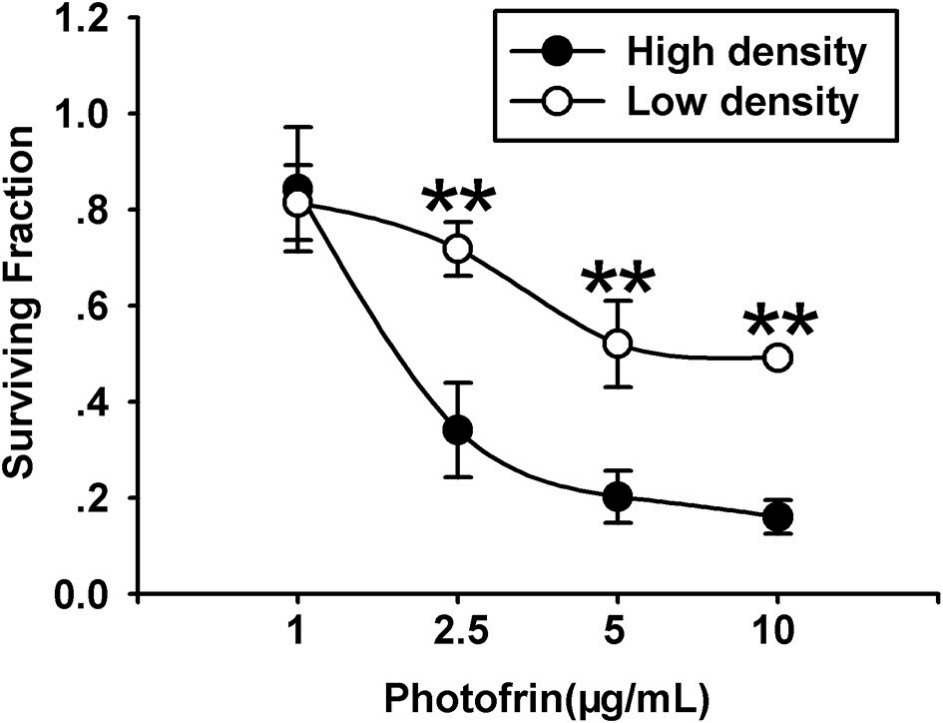
PDT Phototoxicity depends on the cell density. CCK-8 assay was utilized to measure cell survival after PDT under high- or low-density conditions. Data points are mean±SD from 4 to 7 independent experiments. *t-*test was used to assess statistically significant differences between groups. ^**^P < 0.01, significantly different from high-density group.

For exploring the role of Cx40 channels in cellular survival after Photofrin-mediated PDT, Cx40-expression cells were seeded at high and low cellular density. Under the condition of high density (3 × 10^4^cells/cm^2^), the formation of the Cx channel was possible, while at low density (3 × 10^3^cells/cm^2^), cells did not directly contact each other, and no channel was formed. As illustrated in Figure 1, PDT could markedly reduce cell survival in a concentration-dependent manner under conditions of high and low density. It is worth noting that the phototoxic action of PDT was remarkably enhanced at high cellular density, where cells have the opportunity to attach other cells, than that at low cellular density. Specifically, when cells were treated with medium (2.5 μg/mL) and high (5 and 10 μg/mL) concentrations of Photofrin, cell survival under the condition of low density was significantly enhanced when compared to that under high-density condition. Nevertheless, no statistically significant difference in cellular survival was observed between high- and low-density conditions when cells were treated with a low concentration of Photofrin (1 μg/mL). These results indicate that at high Photofrin concentration, the phototoxic action of PDT is enhanced when there are opportunities for forming Cx40 channels.

### Density Dependence of PDT Phototoxicity Is Mediated by Cx40 Channels

**Figure 2:**
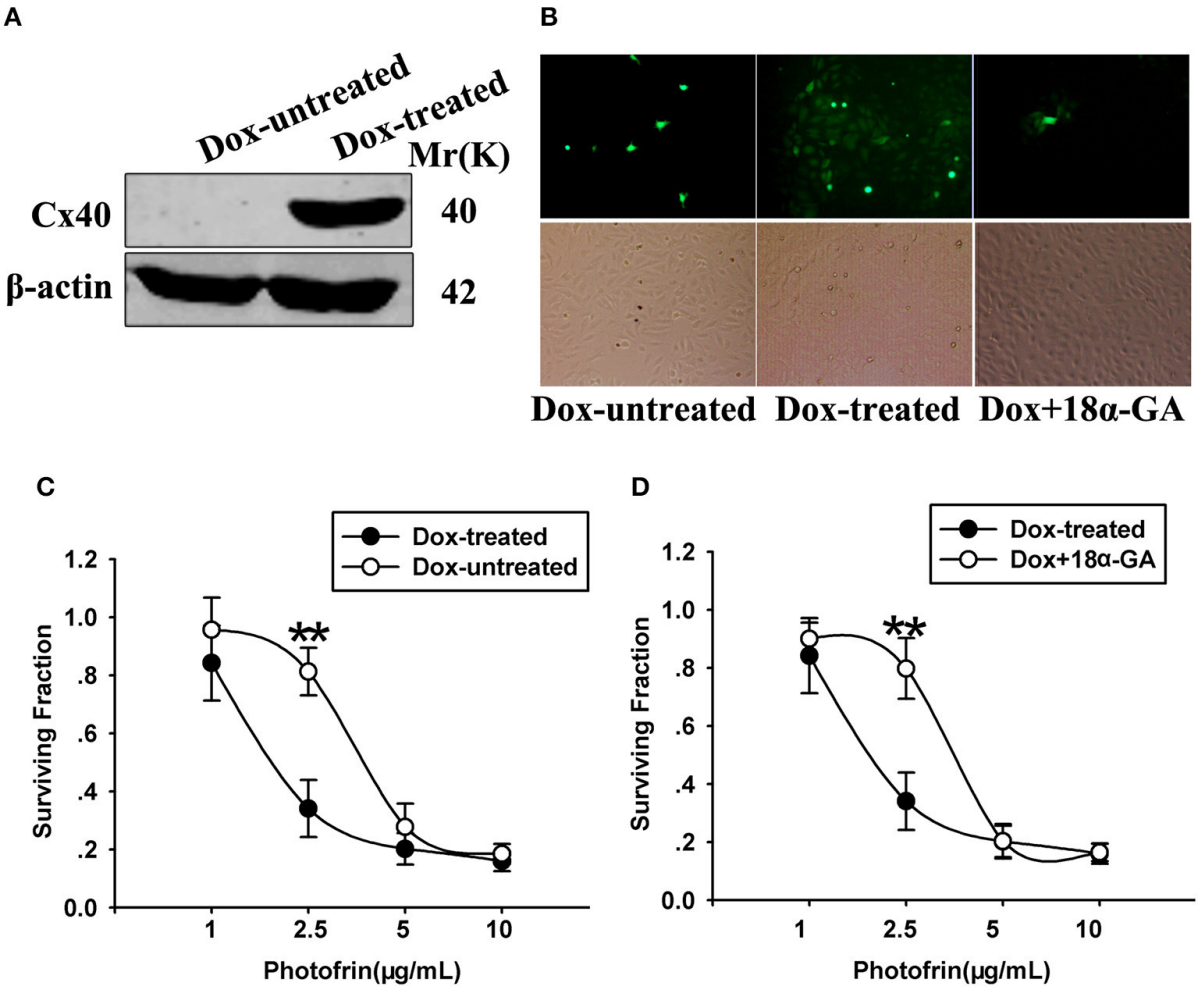
**(A)** Cx40 expression in Dox-treated and -untreated cells was detected by Western blot assay; **(B)** 18α-GA prohibited dye transfer via Cx channels as measured by “parachute” assay. **(C,D)** Cell survival after PDT in cells treated with and without Dox and 18α-GA. Data points are mean±SD from 4 to 7 experiments. *t-*test was used to assess statistically significant differences between groups. ^**^P < 0.01, significantly different from Dox-treated group.

The finding that the phototoxic effect in Cx40-expressing cells was cell density-dependent demonstrates that Cx40 channels might impact PDT phototoxic action. For exploring the role of Cx40-formed channels in PDT sensitivity, Cx coupling was manipulated in the cultures by two methods: chemical inhibition by Cx channel inhibitor and Dox induction of Cx40 expression. As demonstrated in Figure 2A, Dox induced Cx40 expression whereas Cx40 expression was not induced in Dox-untreated cells. Moreover, the function of Cx40 channels in cultured cells was assessed using a parachute dye coupling assay. As depicted in the Materials and Methods section, donor cells were labeled with calcein, a dye penetrating Cx channels, and then incubated with receiver cells. The number of receiver cells containing dye per donor cell was assessed and the function of Cx40 channels was evaluated. As illustrated in Figure 2B, Cx40 channel formation was detected when cells were treated with Dox, whereas in Dox-untreated cells, no channels were observed. After Dox-treated cells were pretreated with 10 μM 18α-GA, the function of Cx40 channels was substantially reduced.

The cell survival of PDT in cells treated with Dox (channel formation) and without Dox (no channel formation) at high cellular density was determined in Figure 2C. After cells were treated with Photofrin at a concentration of 2.5 μg/mL, cell survival in cells treated with Dox (channel formation) was significantly lower than cells without Dox treatment (no channel formation). Specifically, the survival fraction of channel-formed cells was approximately 2.5-fold lower compared to cells without channel formation (Figure 2C). Pretreating channel-formed cells with 10 μM 18α-GA reduced the phototoxic action of Photofrin-PDT, thus resulting in remarkably enhanced survival under the condition of high density (Figure 2D). Specifically, at 2.5 μg/mL Photofrin, 18α-GA-treated cells exhibited lower PDT phototoxicity, with a survival rate approximately 2.5 times higher than for 18α-GA-untreated cells (Figure 2D). Nevertheless, there exhibited no statistically significant difference in cell survival between cells treated with and without 18α-GA, or cells with and without channel formation when cells were treated with 1, 5 and 10 μg/mL Photofrin (Figures 2C,D).

The above data indicate that at medium Photofrin concentration, under high-density conditions, Cx40-formed channel increases the phototoxic effect of Photofrin-PDT as the phototoxic action at high density significantly decreased when Cx40 was not expressed or Cx40-formed channels were suppressed. In short, these results demonstrate that Cx40-formed channels have an ability to increase PDT phototoxicity under high-density conditions.

### Cx40-Formed Channels Increase the Sensitivity of Tumor Xenografts to Photofrin-Mediated PDT

**Figure 3:**
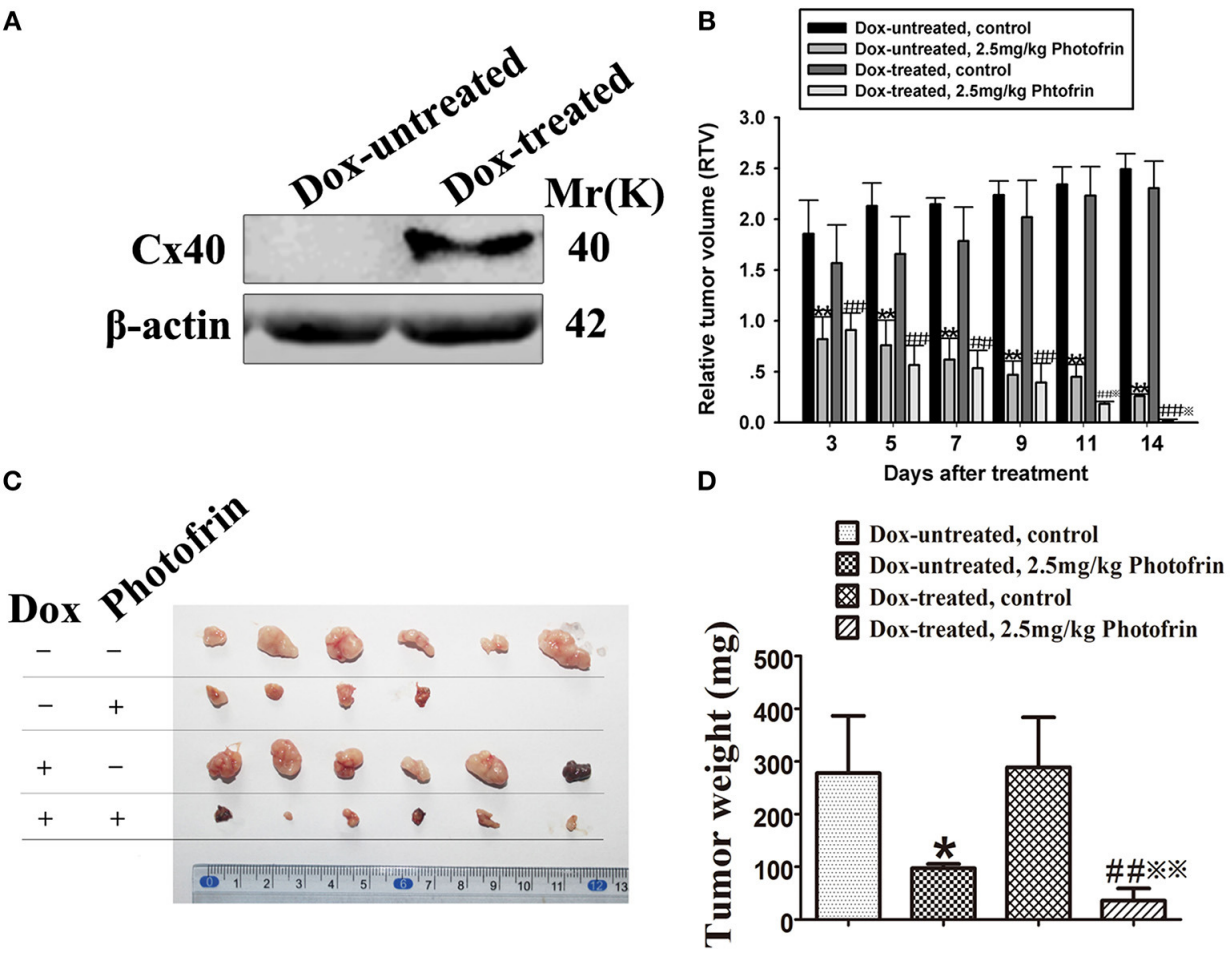
Cx40-formed channels increase the sensitivity of tumor xenografts to Photofrin-mediated PDT. **(A)** Cx40 expression induced by Dox in the xenograft tumors was detected by Western blot; **(B)** The presence of Cx40-composed channels decreases the RTV of xenografts after PDT; **(C,D)** Xenografts' weights after PDT. For **(B–D)** data points are mean ± SD from 4 to 6 independent samples. One-way ANOVA was used to assess statistically significant differences between groups.^*^ *P* < 0.05, ^**^ *P* < 0.01, significantly different from Dox-untreated group; ^##^*P* < 0.01, significantly different from Dox-untreated group; ^※^*P* < 0.05, ^※※^*P* < 0.01, significantly different from 2.5 mg/kg Photofrin group (Dox-untreated).

For exploring the role of Cx40-formed channels in PDT phototoxicity *in vivo*, a xenograft model using nude mice subcutaneously transplanted with HeLa cells transfected with Cx40 was employed. For controlling the expression of Cx40 in tumor xenografts, drinking water with or without Dox was supplied. Before PDT treatment, tumor xenografts in Dox-treated and Dox-untreated groups were randomly selected for detecting Cx40 expression. Figure 3A showed Cx40 expression in the xenografts treated with Dox was detected.

When the tumor xenografts were grown to 100–300 mm^3^, Photofrin or 0.5% sterile dextrose were injected through the tail vein. Twenty-four hours after the injection, the xenografts were irradiated. After PDT, tumor volume was measured and the mean RTV of each group was calculated for assessing tumor growth. The results showed that the tumor growth in xenografts treated with and without Dox was significantly inhibited after Photofrin treatment (Figure 3B). Notably, the mean RTV of Dox-treated xenografts was significantly decreased compared to that of Dox-untreated xenografts after Photofrin-PDT (Figure 3B). Moreover, the Dox-treated mice presented a substantial reduction in tumor weights compared to Dox-untreated mice (Figures 3C,D). The tumor weight inhibitory rates of xenografts treated with and without Dox were 87.54 and 64.99%, respectively (Table 1). The above results suggest that the presence of Cx40-formed channels increases PDT efficacy *in vivo*.

**Table 1:** Tumor weight inhibitory rates after Photofrin-mediated PDT^*^.

**Groups**	**Tumor weight inhibition (%)**
Dox-treated	87.54
Dox-untreateds	64.99

* *Tumor weight inhibitory rates are calculated at the end of treatment as described above*.

### Cx40-Formed Channels Stimulate Intracellular ROS Generation After PDT

**Figure 4:**
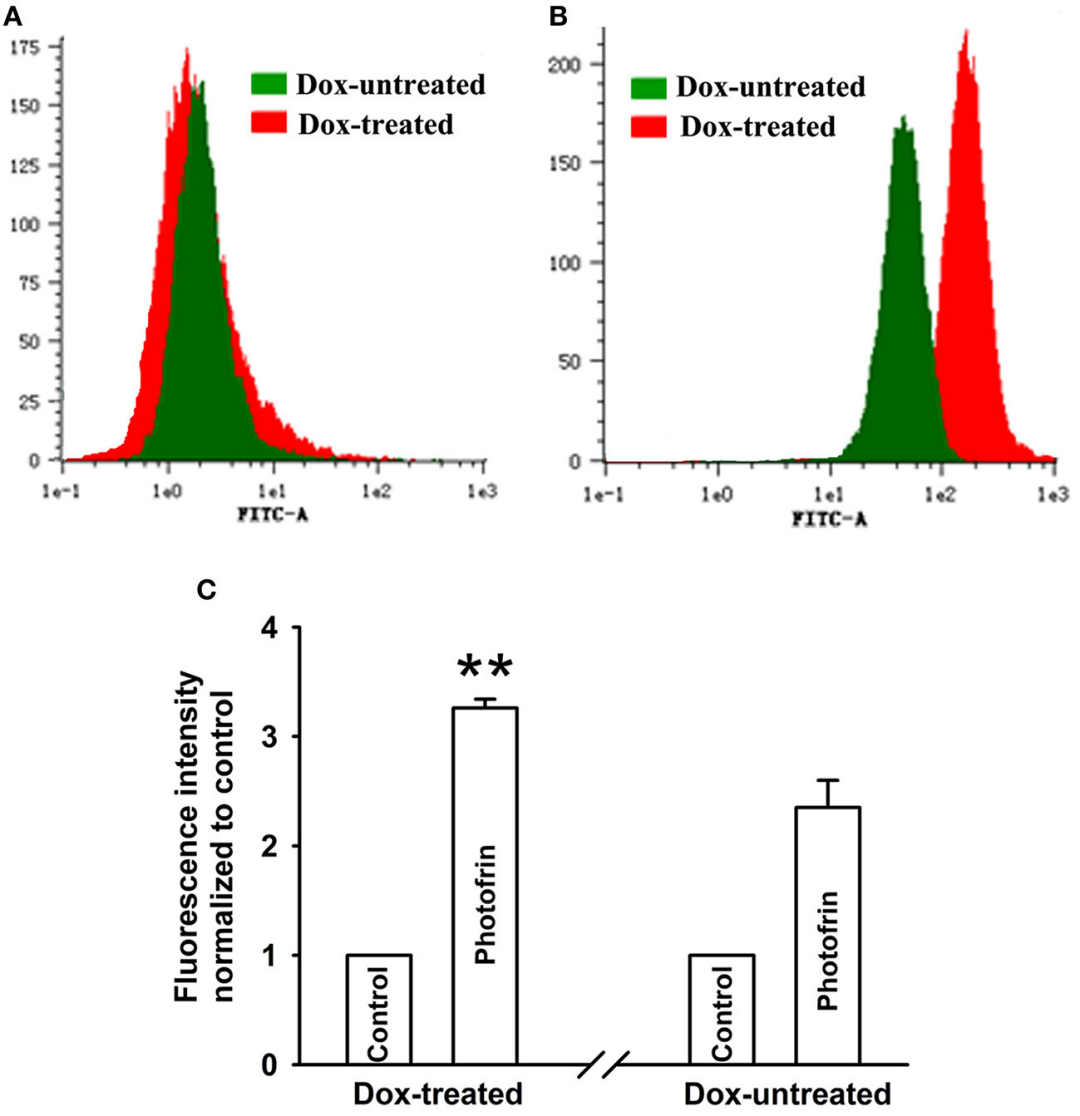
Cx40-formed channels stimulate intracellular production of ROS. After incubation with DCFH-DA, Dox-treated and Dox-untreated cells were irradiated with or without Photofrin. Flow cytometry was performed to measure the fluorescence intensity of ROS 30 min after PDT. **(A)** control; **(B)** 2.5 mg/mL Photofrin; **(C)** The fluorescence intensity of ROS. Data points are mean ± SD from 3 experiments. *t-*test was used to assess statistically significant differences between groups. ^**^ *P* < 0.01, significantly different from Dox-untreated group.

Studies have proven that Cx40 channels allow ROS diffusion despite its short lifetime (11). Therefore, ROS may impact the enhancement of PDT phototoxicity mediated by Cx40 channels. Figure 4 showed that a significantly higher level of intracellular ROS was generated in cells treated with Dox (channel formation) than cells treated without Dox (no channel formation). Specifically, the amount of intracellular ROS in cells treated with Dox increased by a factor of ~1.5 compared to that of Dox-untreated cells. The finding that Cx40-formed channels enhance ROS production indicates that the ROS-mediated pathway may be associated with increased PDT phototoxicity by Cx40-formed channels.

### Cx40-Formed Channels Trigger Ca^2+^ Influx and Release After PDT

**Figure 5:**
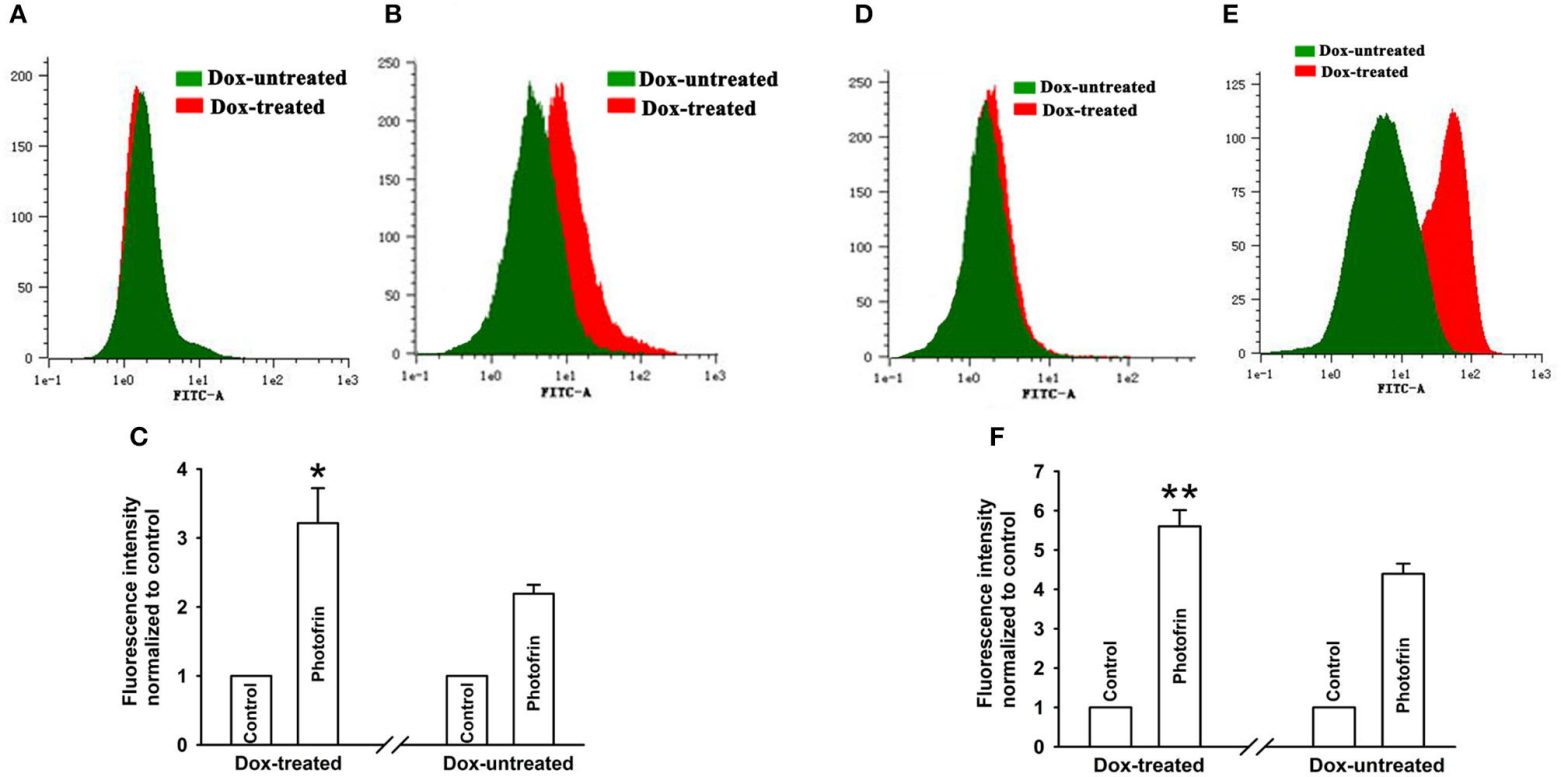
Ca^2+^ release and influx were increased by Cx40-formed channels. After incubation with Fluo-3-Am, Dox-treated and Dox-untreated cells were irradiated with or without Photofrin. Flow cytometry was performed to measure the fluorescence intensity of Ca^2+^ after PDT. **(A)** control; **(B)** 2.5 mg/mL Photofrin; **(C)** The fluorescence intensity of Ca^2+^. For **(A–C)**, the cells were incubated in fresh BBS in the absence of Ca^2+^ during irradiation. **(D)** control; **(E)** 2.5 mg/mL Photofrin; **(F)** The fluorescence intensity of Ca^2+^. For **(D–F)**, the cells were incubated in fresh BBS in the presence of Ca^2+^ during irradiation. Data points are mean ± SD from 3 experiments. *t-*test was used to assess statistically significant differences between groups. ^*^P < 0.05, ^**^P < 0.01, significantly different from Dox-untreated group.

Studies have demonstrated that PDT causes an increment of intracellular Ca^2+^ concentration ([Ca^2+^]_i_) due to the release from intracellular Ca^2+^ store and Ca^2+^ influx from the extracellular medium, thereby inducing apoptosis and cell death (19, 20). Since Ca^2+^ can transfer through Cx channels (19), the presence of Cx channels may stimulate Ca^2+^ release and influx. For determining the role of Cx40-formed channels in Ca^2+^ influx, cells were kept bathing in fresh Ca^2+^ balanced salt solution during irradiation As shown in Figures 5A–C, the amount of [Ca^2+^]_i_ of Dox-treated (channel-formed) cells significantly increased compared to Dox-untreated (channel-unformed) cells, indicating that Cx40-formed channels stimulate the influx of Ca^2+^ from the extracellular medium after PDT. For exploring the role of Cx40-composed channels in Ca^2+^ release after PDT, cells in Ca^2+^-free balanced salt solution were irradiated. As illustrated in Figures 5D–F, the quantities of [Ca^2+^]_i_ of cells treated with Dox (channel formation) were significantly enhanced compared to cells treated without Dox (no channel formation), suggesting that Cx40-formed channels promote intracellular Ca^2+^ release after PDT.

### Cx40-Formed Channels Have No Effect on 4-HNE and Ceramide Generation After PDT

**Figure 6:**
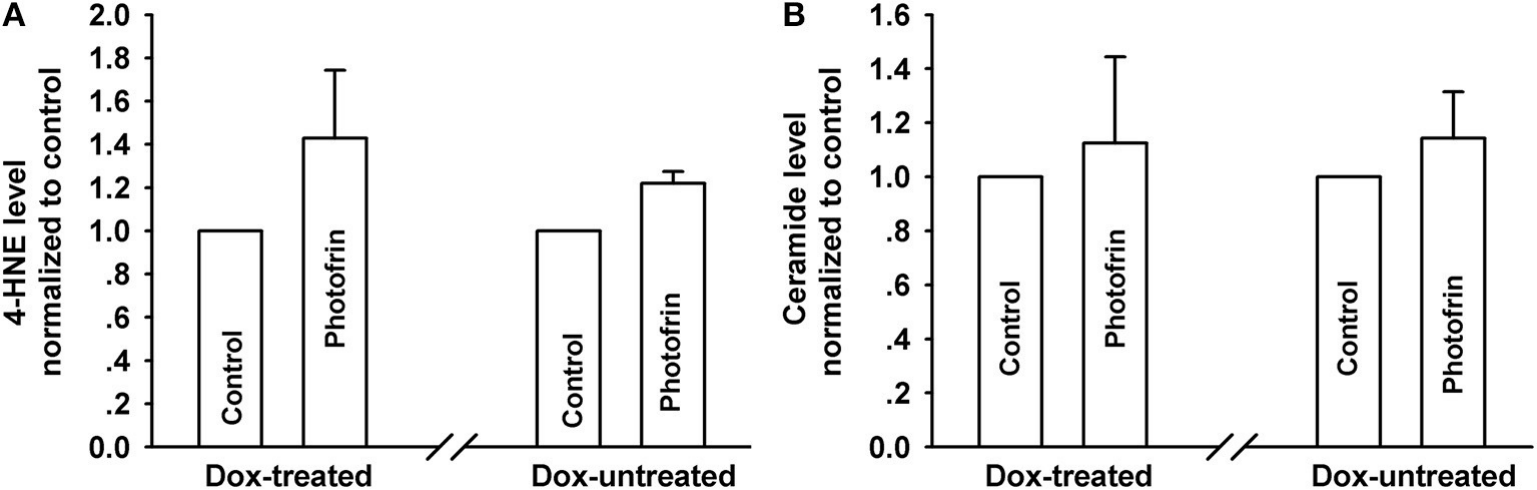
Effects of Cx40-formed channels on 4-HNE and ceramide production after PDT. Cells were pretreated with or without Dox, then irradiated with or without Photofrin. The levels of 4-HNE and ceramide were detected using ELISA assay. **(A)** 4-HNE; **(B)** ceramide. For **(A,B)**, data points are mean ± SD from 3 experiments. t-test was used to assess statistically significant differences between groups.

PDT induces the accumulation of intracellular lipid peroxides, among which 4-HNE and ceramide might diffuse via Cx channels due to their small molecular weights (13, 21). Thus, these lipid peroxides may be responsible for the enhanced phototoxicity of PDT by channels formed by Cx40. However, there exhibited no statistically significant difference in the amounts of 4-HNE and ceramide, indicating that intracellular 4-HNE and ceramide were did not account for the enhanced phototoxicity of PDT by Cx40-formed channels (Figure 6).

## Discussion

Although lack of Cxs or Cx channels in many tumors is generally associated with tumorigenic phenotype, the expression of Cxs can be maintained or up-regulated in some cases. Reports have shown that in testicular tumors, Cx40 expression is increased when compared with the testis (22), and in small lung cancer, Cx40 expression is maintained (23). In these cases, Cx40-formed channels would be expected to have a role in the efficacy of antitumor therapy.

Our previous studies demonstrated that a substantial increase of PDT efficacy depended on Cx channels composed of Cx32 and Cx26 among the target cells (11, 12). The present study shows that Cx40-formed channels hold the potential to enhance PDT phototoxicity. This action is absent under low-density conditions owing to the lack of channel formation (Figure 1). It should be noted that under high-density conditions, where there is a chance for the cells to contact with each other and form Cx40 channels, inhibition of Cx40-composed channels either by suppressing the expression of Cx40 or by pretreating with 18α-GA, a Cx channel inhibitor, attenuates the phototoxicity of PDT (Figures 2C,D). Additionally, the presence of Cx40-composed channels enhanced the phototoxicity of PDT in the tumor xenografts (Figures 3B–D; Table 1). This study illustrates that enhancing the function of Cx40 channels may increase the sensitivity of tumor cells to PDT and suggests that treatment strategies designed to increase the expression of Cx40 or to sustain the functionality of Cx40 channels might increase PDT treatment outcome.

It should be noted that several analgesics, such as tramadol and flurbiprofen, have been shown to inhibit the function of Cx channels (24). Thus, if PDT is used concurrently with analgesic treatment for patients accompanying carcinous pain, the antineoplastic efficiency of PDT may be reduced by the inhibition of Cx channels caused by analgesics. It has also been noted that several agents, such as simvastatin and baicalein, have been reported to enhance the functionality of Cx channels, leading to an increase in the efficacy of antineoplastic agents by enhancing toxic bystander effects (25, 26). Therefore, the efficacy of PDT for tumors with Cx channels can be enhanced in the event of PDT used concurrently with these agents in clinical settings.

It is worth noting that the effects dependent on Cx40 channels on phototoxicity were absent at high (such as 5 and 10 μg/mL) and low (such as 1 μg/mL) Photofrin concentrations in the study (Figures 2C,D). This indicates that the Cx channel-dependent effect on PDT efficacy depends on the concentration of the photosensitizer clinically used. At low concentrations, the diffusion of toxic substances mediated by Cx40 channels after PDT can only kill a small fraction of malignancies and a Cx channel-dependent component of PDT phototoxicity would be weakened. At high concentration, almost all cells were directly killed and thus Cx channels would not affect PDT phototoxicity. Nevertheless, at the clinically proper Photofrin concentration used, Cx40 channels may improve PDT phototoxicity by augmenting intercellular diffusion of toxic products, and one should note that maintaining or increasing the function of Cx40 channels may increase the sensitivity of tumor cells to PDT.

It is generally believed that the “bystander effect” of Cx channels depends on the diffusion of toxic signals from one cell to neighboring cells through Cx channels (27). Reports have proven that PDT-induced ROS generation results in cell injury by assaulting the integrity of biomolecules such as DNA, lipids and proteins (28), and intracellular ROS can propagate Cx complexes as a signal of oxidative stress (28). Hence, the Cx40-formed channel-dependent effect of PDT-induced cytotoxicity may be associated with intracellular ROS transfer via Cx channels. The results indicated that the quantity of intracellular ROS induced by PDT in Dox-treated (channel formation) cells was significantly enhanced compared to in Dox-untreated (no channel formation) cells (Figures 4A–C), suggesting that the transfer of intercellular reactive oxygen species may account for the increase induced by Cx40-formed channels in PDT phototoxicity. It has been reported that mitochondrial membrane damage by PDT induces the increase in ROS generation, contributing to the damaging effect of PDT (29). Thus, mitochondrial-dependent oxidative stress may be involved in the increase of PDT phototoxicity by Cx40 channels. The contribution of mitochondria-mediated oxidative stress to the enhancement of phototoxicity by Cx40 channels should be investigated in further studies.

It has been documented that PDT can induce intracellular Ca^2+^ release from calcium stores and extracellular Ca^2+^ influx, causing the enhancement of [Ca^2+^]_i_, ultimately resulting in cellular death and apoptosis (20). Studies have been indicated that Ca^2+^ was allowed to propagate via Cx channels to unexposed neighbors (30). Thus, the diffusion of Ca^2+^ through Cx channels may have impacts on PDT photocytoxicity. Nevertheless, reports have indicated that the enhancement of [Ca^2+^]_i_ may result in Cx coupling closure (31). It is widely believed that the [Ca^2+^]_i_ impacting Cx channels is dependent on the species of cell and Cx expressed (32). The results that the increment of intracellular Ca^2+^ concentration was mediated by Cx40-formed channels suggested that the increase in intracellular Ca^2+^ concentration induced by PDT has no impact on Cx40-formed channels (Figures 5A–F). More importantly, the findings demonstrated that Cx40-formed channels contributed to the enhancement of Ca^2+^ influx from the extracellular medium and Ca^2+^ release from intracellular Ca^2+^ store by PDT (Figures 5A–F). Taken together, we can conclude that the increment of PDT-induced phototoxicity by Cx40-formed channels may be related to the intracellular Ca^2+^ pathway. Nevertheless, whether the intracellular Ca^2+^ pathway is required for the increment of phototoxicity mediated by Cx40 channels needs to be investigated in further studies.

It has been established that Ca^2+^ is allowed to propagate through Cx40-formed hemichannels, contributing to ATP release in juxtaglomerular endotheliocytes (33). Thus, Cx40-mediated enhancement of intracellular Ca^2+^ concentration may be partially attributed to Ca^2+^ influx via hemichannels composed by Cx40, which may contribute to Cx40-mediated increment of PDT phototoxicity. However, the role of hemichannels in oncotherapy including PDT has been less reported. It would be interesting to uncover the role of hemichannel in cancer treatment and its underlying mechanisms in further study.

It has been established that PDT can cause an increment of lipid peroxide production, which may be responsible for PDT-induced apoptosis and cell death (14). Among these lipid peroxides, 4-HNE and ceramide may diffuse via Cx channels since their molecular weights are less than the upper limit (<1.5 kDa) of penetrable molecules via Cx channels. Thus, these lipid peroxides may account for the increase in PDT-induced phototoxicity. However, the results showed that the levels of 4-HNE and ceramide exhibit no significant difference between cells with and without Cx40 channels (Figures 6A,B). The findings are inconsistent with our previous results, which demonstrated that Cx26-formed channels contributed to the increase of 4-HNE and ceramide production after PDT (11). It should be noted, however, that the biophysical permeation properties of penetrable substances depend on the nature of the Cx species forming channels. For example, cAMP and cGMP were allowed to diffuse through homogeneous Cx26 channels, whereas cAMP was not permitted to propagate via heterogeneous Cx26/Cx32 channels (34, 35). The results that Cx40 channels do not affect 4-HNE and ceramide generation suggest that lipid peroxide-mediated signaling pathways may not be involved in the enhanced PDT efficacy by Cx40-formed channels (Figures 6A,B).

Collectively, the study demonstrates the increased effects of Cx40-formed channels on PDT-mediated oncotherapy and suggests a key insight in considering bystander effects and intercellular signaling via Cx40-formed channels in response to PDT-mediated cancer treatment. The function of Cx40-formed channels in human malignant tumors may be an important determining factor of the response to PDT-mediated oncotherapy in clinic, which brings about a few therapeutic considerations. First of all, maintenance or even transitory enhancement of Cx40 expression and Cx40-formed channels is a beneficial strategy for increasing the therapeutic effect of PDT. Oppositely, factors inhibiting the function of Cx40-composed channels may cause a decrease in the sensitivity of malignancies to PDT, resulting in a significant decline in the therapeutic effect of PDT.

## Ethics Statement

This study was carried out according to the principles of the Basel Declaration and recommendations of Guidance Suggestions for Caring for Laboratory Animals, Animal ethics committee of Xuzhou Medical University. The protocol was approved by the Animal Ethics Committee of Xuzhou Medical University.

## Data Availability

The datasets for this manuscript are not publicly available because of data confidentiality. Requests to access the datasets should be directed to Professor Xiao-Xing Yin, yinxx@xzhmu.edu.cn.

## Author Contributions

X-XY and D-PW designed the experiments. D-PW, L-RB, Y-FL, YZ, C-HD, S-MY, FZ, J-LH, and Y-YW performed the experiments. D-PW, L-RB, and Y-FL analyzed the data. D-PW wrote the paper. D-PW, L-RB, Y-FL, and YZ contributed analysis tools, reagents, and materials.

### Conflict of Interest Statement

The authors declare that the research was conducted in the absence of any commercial or financial relationships that could be construed as a potential conflict of interest.
