# Corrigendum: A Novel Role of Connexin 40-Formed Channels in the Enhanced Efficacy of Photodynamic Therapy

**DOI:** 10.3389/fonc.2022.853278

**Published:** 2022-02-23

**Authors:** Deng-Pan Wu, Li-Ru Bai, Yan-Fang Lv, Yan Zhou, Chun-Hui Ding, Si-Man Yang, Fan Zhang, Yuan-Yuan Wang, Jin-Lan Huang, Xiao-Xing Yin

**Affiliations:** ^1^ Jiangsu Key Laboratory of New Drug Research and Clinical Pharmacy, Pharmacy School of Xuzhou Medical University, Xuzhou, China; ^2^ Department of Pharmacology, Pharmacy School of Xuzhou Medical University, Xuzhou, China; ^3^ Department of Pharmacy, Wuxi Ninth Affiliated Hospital of Suzhou University, Wuxi, China; ^4^ Scientific Research Center of Traditional Chinese Medicine, Guangxi University of Chinese Medicine, Nanning, China

**Keywords:** Connexin 40, channel, photodynamic therapy, reactive oxygen species, calcium

In the original article, there was a mistake in **Figure 5** as published. After confirmation, we found that the representative images of the flow cytometer of Dox-untreated and Dox-treated control group (panel A) and Photofrin group (panel E) in **Figure 5** were misused due to our carelessness in the selection of representative images for image combination using software of flow cytometer. The corrected **Figure 5** appears below.

**Figure 5:**
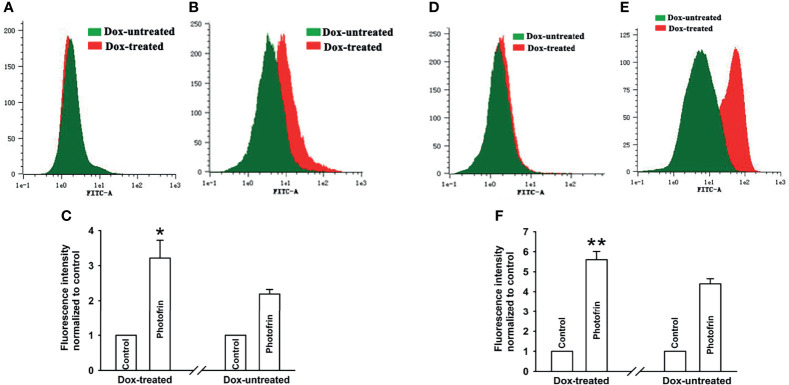
Ca^2+^ release and influx were increased by Cx40-formed channels. After incubation with Fluo-3-Am, Dox-treated and Dox-untreated cells were irradiated with or without Photofrin. Flow cytometry was performed to measure the fluorescence intensity of Ca^2+^ after PDT. **(A)** control; **(B)** 2.5mg/mL Photofrin; **(C)** The fluorescence intensity of Ca^2+^. For **(A–C)**, the cells were incubated in fresh BBS in the absence of Ca^2+^ during irradiation. **(D)** control; **(E)** 2.5mg/mL Photofrin; **(F)** The fluorescence intensity of Ca^2+^. For **(D–F)**, the cells were incubated in fresh BBS in the presence of Ca^2+^ during irradiation. Data points are mean ± SD from 3 experiments. *t* test was used to assess statistically significant differences between groups. *P < 0.05, **P < 0.01, significantly different from Dox-untreated group.

 The authors apologize for this error and state that this does not change the scientific conclusions of the article in any way. The original article has been updated.

## Publisher’s Note

All claims expressed in this article are solely those of the authors and do not necessarily represent those of their affiliated organizations, or those of the publisher, the editors and the reviewers. Any product that may be evaluated in this article, or claim that may be made by its manufacturer, is not guaranteed or endorsed by the publisher.

